# Role of Three-Dimensional Printing in Treatment Planning for Orthognathic Surgery: A Systematic Review

**DOI:** 10.7759/cureus.47979

**Published:** 2023-10-30

**Authors:** Manaf O Alhabshi, Haya Aldhohayan, Olla S BaEissa, Manar S Al Shehri, Nawaf M Alotaibi, Sarah K Almubarak, Abdulrahman A Al Ahmari, Hayithm A Khan, Hesham A Alowaimer

**Affiliations:** 1 Oral and Maxillofacial Surgery, King Abdullah Medical City, Jeddah, SAU; 2 Dentistry, Buraydah Colleges, Buraydah, SAU; 3 General Dentistry, North of Riyadh Dental Clinic, Second Health Cluster, Riyadh, SAU; 4 General Dentistry, Ibn Sina National College, Jeddah, SAU; 5 Dentistry, Dar Al Uloom University, Riyadh, SAU; 6 Vision College, King Abdulaziz University, Jeddah, SAU; 7 Orthodontics, Ministry of Health, Riyadh, SAU; 8 College of Dentistry, Imam Abdulrahman Bin Faisal University, Dammam, SAU; 9 Oral and Maxillofacial Surgery, Ministry of Health, Jeddah, SAU; 10 Maxillofacial Surgery, Ministry of Health, Dhahran, SAU

**Keywords:** lefort i osteotomy, bilateral lefort ii osteotomy, orthodontic surgery, digital dentistry, oral and maxillofacial surgeon

## Abstract

Three-dimensional (3D) printing refers to a wide range of additive manufacturing processes that enable the construction of structures and models. It has been rapidly adopted for a variety of surgical applications, including the printing of patient-specific anatomical models, implants and prostheses, external fixators and splints, as well as surgical instrumentation and cutting guides. In comparison to traditional methods, 3D-printed models and surgical guides offer a deeper understanding of intricate maxillofacial structures and spatial relationships. This review article examines the utilization of 3D printing in orthognathic surgery, particularly in the context of treatment planning. It discusses how 3D printing has revolutionized this sector by providing enhanced visualization, precise surgical planning, reduction in operating time, and improved patient communication. Various databases, including PubMed, Google Scholar, ScienceDirect, and Medline, were searched with relevant keywords. A total of 410 articles were retrieved, of which 71 were included in this study. This article concludes that the utilization of 3D printing in the treatment planning of orthognathic surgery offers a wide range of advantages, such as increased patient satisfaction and improved functional and aesthetic outcomes.

## Introduction and background

Orthognathic surgery, also referred to as corrective jaw surgery, is a medical procedure that consists of a series of operations performed on the jaw and jawline to alter and/or enhance the facial features [1]. In 1849, Simon P. Hullien performed the first mandibular osteotomy to surgically correct prognathism and classify malocclusion as class III [2]. The orthodontics field has immensely evolved over time, with orthognathic surgery broadening its scope beyond malocclusion correction and facial aesthetics [3]. Today, orthognathic surgeries are performed for a variety of reasons such as correcting functional issues, birth defects, traumatic injuries, facial asymmetry, orthodontic treatment, and malocclusions [3-5].

The success of orthognathic surgery depends on the ability to comprehend and articulate the patient’s wishes, match them to the diagnosis, and formulate and execute a treatment plan accurately. Preoperative prediction and clinical examination are essential parts of orthognathic surgical planning. The surgical plan depends not only on the bone and dental diagnosis but also on the presurgical prognosis. To achieve the desired outcome, careful coordination between orthodontists and surgeons is important during all stages of treatment [6]. When orthognathic surgery is performed, the surgeon must first determine the initial dentoskeletal relationship, then determine the intended final position, and finally create a three-dimensional (3D) model of the movements required to achieve the goal [7,8]. The primary treatment objectives are establishing orofacial function, achieving facial aesthetics, considering the patency of the airway, and making sure the results are consistent. The systemic clinical examination is subdivided into five primary examinations, namely, the profile view, the frontal view, the three-quarter view, the temporomandibular joint examination, and the occlusal evaluation [7,8].

The traditional orthognathic surgical practice consists of the collection of multiple data points, the implementation of a mock surgical procedure, and the subsequent execution of the same surgical procedure in the operating theater. It also includes cephalometric radiographs with trace elements, facial photographs, and dental impressions. The goal of each step is to create a representative model of the current relationship between the maxilla/mandible and the dental skeletal dysplasia associated with it. This relationship is then used to model surgery to evaluate the potential jaw movements and directly create surgical guide splints, which are essential for the precise intraoperative placement of the maxilla or mandible [9,10]. This kind of surgery uses a traditional analytical model that takes the numbers and transfers the expected 3D movements right to the patient so they can figure out where to place the maxilla or mandible during the surgery [10]. This approach, however, requires a lengthy analytical and radiographic procedure, as well as the development of dental models and splints, which takes a long time and a solid understanding of dental materials and may result in greater miscalculations during the algorithmic stage [10].

Orthognathic surgical procedures have transformed dramatically with the advent of the digital revolution. Computer-aided surgical planning allows surgeons to design the whole procedure on a computer before carrying it out. It creates a virtual representation of the patient’s face and skull using cutting-edge imaging technology such as CT scanners and 3D modeling [11]. Surgical navigation systems are utilized during surgery to give the surgeon real-time tracking. Infrared cameras, trackers, and computer algorithms are used to monitor the placement and movement of surgical equipment as well as the patient’s anatomy. This aids in the maintenance of appropriate jaw posture and alignment and lowers the possibility of surgical mistakes [12]. Intraoral scanners, cone-beam computed tomography (CBCT), and other relevant imaging technologies can be employed to provide real-time visualizations of the patient’s anatomy. These illustrations assist the surgeon in calculating surgical movement accuracy and making necessary changes [13,14].

Additive manufacturing, also known as 3D printing, is the course of adding layers of material to a particular digital design to form 3D shapes and structures. It is a technique that allows the production of high-precision shapes and structures [15]. The increasing demand for products with a wide variety of designs and applications paved the way for the emergence of 3D printing and the development of the fourth industrial revolution. The utilization of 3D technology has enabled considerable progress in a variety of medical treatments and surgical procedures [16-18].

3D printing has been attracting a lot of attention lately as a way to improve intraoperative accuracy during orthognathic procedures. It allows virtual preoperative simulation and enables the creation of personalized bone fixation and bone reconstruction materials. It also helps in creating customized surgical guides and surgical planning by physical models and templates. The use of 3D printing has also contributed to the development of surgical education and improved physician-patient relationships. This review provides an overview of the most recent developments in the utilization of 3D printing in orthodontic surgery, as well as insights into treatment planning in orthognathic surgeries. The research is done based on the question: can surgical outcomes in orthognathic surgery be significantly improved with treatment planning involving 3D printing?

## Review

Methodology

Literature Search

**Figure 1:**
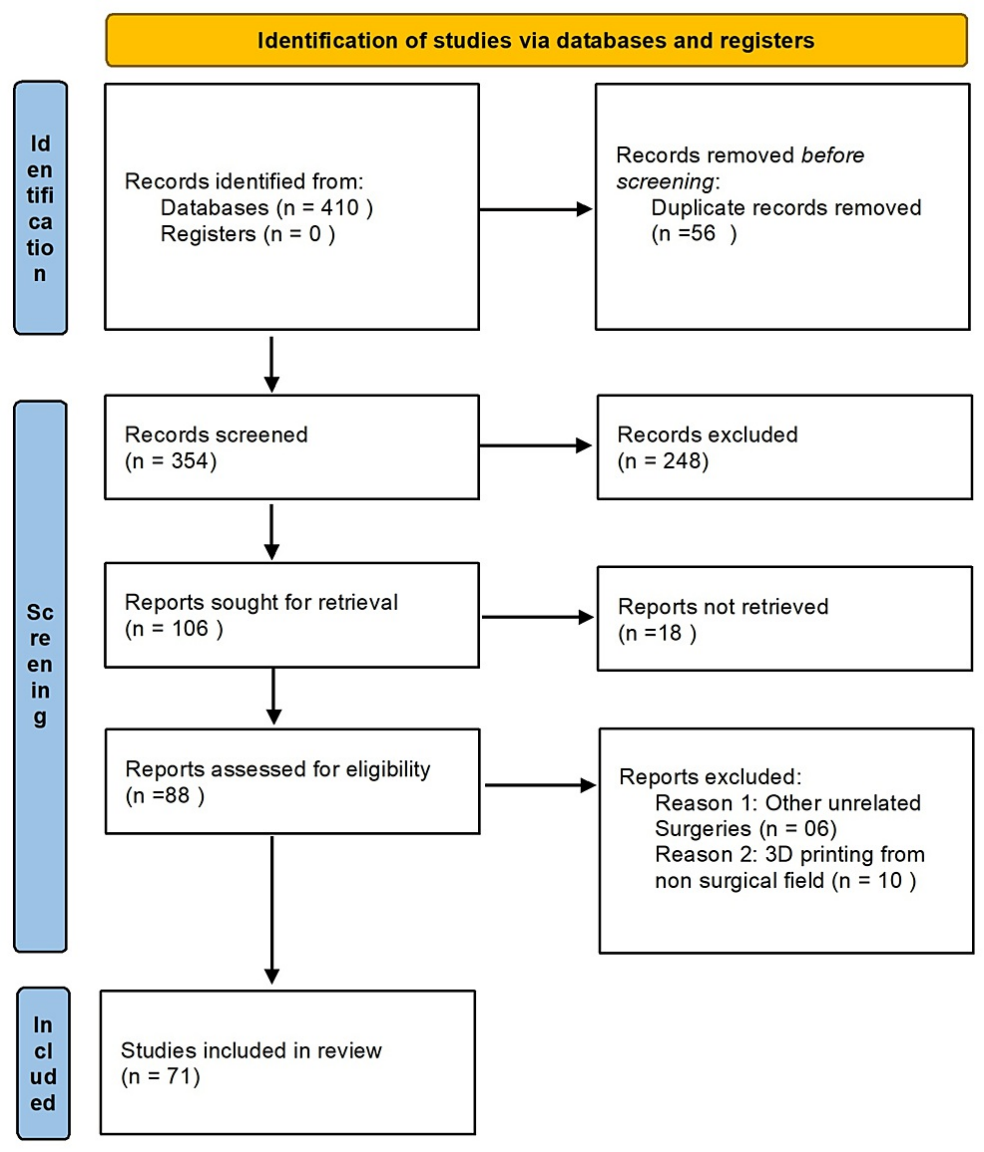
Preferred Reporting Items for Systematic Reviews and Meta-Analyses 2020 flow diagram.

To retrieve relevant articles, PubMed, Google Scholar, ScienceDirect, and Medline databases were searched with relevant keywords. Studies published from 2010 to 2023 were searched using various keywords such as 3D printing, three-dimensional printing, 3D printing in orthognathic surgeries, use of 3D printing in the treatment planning of orthognathic surgeries, computer-aided manufacturing in orthognathic surgeries, and clinical trials on 3D printing in orthognathic surgeries. A total of 410 articles were retrieved, of which 71 articles published from 2010 to 2023 were included in this study (Figure 1).

Eligibility Criteria

Studies with the use of 3D printing in orthognathic surgery were searched. Special attention was paid to studies that included the use of 3D printing in treatment planning during orthognathic surgery. A number of articles were excluded based on the criteria listed below in Table 1.

**Table 1:** Exclusion criteria.

Exclusion criteria	Remarks
Non-human studies	
Non-English studies	
Simple review articles	Meta-analysis was relevant and if it presented a conclusion on clinical trials
Not relevant to 3D printing	
Not relevant to orthognathic surgery	
Correspondences and commentaries	
Material sciences-related studies	

Results

A total of 410 articles were retrieved, of which 71 published from 2010 to 2023 were included for full-text analysis discussing the applications of 3D printing in treatment planning of orthognathic surgeries. A detailed analysis is presented below.

Is Traditional Treatment Planning Still Relevant?

Traditional surgical planning (TSP) involves a two-dimensional (2D) analysis of cephalometry, and dental casts affixed to the articulator, with a facial bow transfer of the occlusal plane of the patient. To define a treatment objective and generate a surgical plan, diagnostic data collected from clinical and radiographic preoperative evaluations and model analysis are combined. Surgeons also use manual model surgery to predict the direction and degree of displacement in the jawbone segment [19,20]. The end of the 20th century has marked a rapid rise in the development and utilization of 3D technology, including computer-aided design (CAD)/computer-aided manufacturing (CAM) and 3D computer-aided design systems, which has led to considerable innovation in the field of orthosurgical planning.

Although there are limitations in TSP, especially regarding treatment planning for complex dentofacial deformities, it has become the standard procedure over the years of trial and error [21]. In a recent meta-analysis, Chen et al. reviewed several randomized clinical trials to investigate the effectiveness of TSP in comparison to virtual surgical planning (VSP) in orthognathic surgeries [22]. The study concluded that both TSP and VSP had similar surgical accuracy when the surgeries were performed on hard tissues in a sagittal plane. In soft tissues, however, VSP showed more promising outcomes. Both VSP and TSP demonstrated a significantly greater surgical accuracy for the maxilla compared to the mandible. In specific regions such as the anterior part of the maxilla, VSP was more accurate in comparison to TSP. Patients who were treated with VSP had better symmetrical frontal view than those treated with TSP [22].

In another study, Barone et al. studied the comparative accuracy of jaw repositioning using digital and traditional surgical planning in bimaxillary orthognathic surgeries of skeletal class III patients. In their reports, digital surgical planning demonstrated a significantly better precision of jaw repositioning compared to the conventional procedure [23]. Studies have shown that the incorporation of additional data can significantly enhance treatment planning precision, especially in facial asymmetry cases such as in cleft lip/palate patients. A prospective study in which 30 patients with cleft lips were enrolled for two-jaw, single-splint orthognathic surgery revealed that transferring 2D orthodontic surgery plans into a 3D setting significantly improved the treatment planning accuracy and treatment outcomes [24].

Based on the above-mentioned findings, it can be concluded that traditional 2D orthodontic surgical planning techniques remain applicable. Despite the continued popularity of traditional 2D approaches for planning orthognathic surgery, the use of 3D simulation is steadily expanding.

Overview of 3D Modeling

The process of 3D modeling involves the fabrication and reconstruction of a virtual 3D representation of a physical object or surface from imaging data [25]. This technology has enabled the transformation of 2D data into 3D data [26]. Traditionally, this method has been used in the manufacturing industry, but it is now used in the medical and dental fields, as well as in plastic surgery and orthodontic surgery. 3D-printed models in the medical field are used for a wide range of applications, including accurate modeling of anatomy and pathology to support preoperative design and simulation of complex surgical or intervention procedures [27]. In the medical field, one of the benefits of this technique is that it gives clinicians a hands-on approach that allows them to evaluate patient anatomy and plan surgeries without having to see the patient in person [28].

Patient-specific 3D models are typically created through the utilization of the patient’s CT scans, MRI, X-rays, or 3D ultrasound images, which are processed and segmented to extract the intended anatomical regions and pathology from the volume images. Segmentation of the images is necessary to separate the subjects of interest and generate the 3D model [29]. The multi-part 3D models are transformed into a series of surface mixtures and prepped for 3D printing by incorporating connectors and surface color data [30]. Commercial software packages such as Mimics MeVislab and Analyze are widely used to process and segment images for 3D printing. Some open-source tools such as 3D Slicer and ITK-SNAP are also used to develop medical models for various clinical applications [31].

An increasing number of studies in the literature suggest that 3D printing models can be accurately replicated and developed for a wide variety of clinical applications [32-34]. In a case study, Mathew et al. reported the clinical benefits of 3D models in surgical planning and execution. In treating mid-face deficiencies, the use of a preoperatively bent reconstruction plate resulted in improved outcomes and improved patient satisfaction [35]. In another study, Narita et al. compared the length of time it took to operate on 25 patients who had a 3D model used in preoperative simulations and 20 patients who did not have a 3D model. The results demonstrated significantly different operating times between the two groups [36]. Another study was conducted to evaluate the 3D printing technology in the treatment planning of complex maxillofacial procedures. According to the results, 3D models not only significantly improved the predictability but also the treatment outcomes. Using 3D models, the duration of the operation was shortened, resulting in a shorter period of general anesthesia and a shorter period of wound exposure [37].

3D Printing and Pretreatment Planning of Orthognathic Surgery

3D-printed models and surgical guides for presurgical planning: Treatment planning refers to a process in which fundamentally relevant clinical information is collected to decide the best options that are efficient, accurate, and save operation time. Pre-planning is key in several aspects, especially to reduce risks and spend less time in the surgical suite [38]. The process of preoperative planning involves the careful analysis of medical images and other characteristics of patient information to gain better insights into the current problem and construct a model suitable for the patient [39]. All surgical subspecialties have been employing 3D-printed models for presurgical planning. These models allow accurate planning and simulation of surgical procedures, incisions, and placement and sizing of required hardware so there is no need to perform these steps intraoperatively [40]. Moreover, accurate and realistic models can be produced that provide interpretable visual guides [41].

Multiple studies have reported the efficiency of 3D printing for better preoperative planning. It is reported to considerably improve surgical outcomes by decreasing postsurgical morbidity, surgeon performance, duration of surgical procedures, less exposure to ionizing radiations, and other aspects of overall learning [42]. Recent advances in computer-aided preoperative planning have revamped the analysis of surgical planning and offered a better presentation of the craniofacial complex which has enhanced the predictability of surgical outcomes [43].

3D printers have revolutionized the way we make orthopedic splints and changed the way we treat temporomandibular joint conditions. A study was conducted by Ye and colleagues in which digital splints designed using a Boolean operation were applied to various offset models modified through CAD software. The study revealed that offset dental models are more advantageous for the use of 3D-printed splints, as they are more capable of adhering to teeth [44]. After reporting a lower rate of errors compared to prior studies, Shaheen et al. recommended the clinical utilization of 3D endoscopic occlusion splints [45]. A few years after the initial publication of the study, a new research paper was published on 3D orthognathic splints. The study produced clinically acceptable results and was reproducible, and it was concluded that the protocol could be applied to the design and fabrication of intermediate splints for bimaxillary orthognathic surgery [45].

In planning orthognathic surgery, preoperative planning is the most critical part of the procedure. Traditional 2D technologies used in the diagnosis, planning, and fabrication of splints present limitations for orthognathic surgical planning as they cannot provide 3D information on anatomical structures. Moreover, inaccuracies may arise due to low-resolution related issues which are transferred to the design of suboptimal plaster cast [46]. These shortcomings were overcome by the incorporation of 3D printing in orthognathic procedures which provides high-resolution imaging to ensure accurate skeletodental models and splints when transferring anatomical landmarks. 3D printing also ensures low radiation exposure and considerable accuracy in recording the anatomy of patients via high-resolution imaging. This improves the repositioning of jaws in a computerized workflow [16].

Preoperative 3D imaging such as CT and CBCT are accurate volumetric techniques along with 100-200 µm voxels of spatial resolution which accurately deliver anatomical features of patients. These are then transferred to suitable planning platforms [16]. These images are used to build various 3D-printed objects such as occlusal splints, anatomical models, patient-specific implants, and cutting guides [47]. 3D-printed surgical guides help in cutting bones, as well as placement of implants, and enable the surgery with maximum accuracy and minimal invasive involvement [26]. 3D-printed guiding splints of the jaw bones specific to the patient exactly replicate their original form and function providing an exact fit for the graft [47]. 3D-printed appliances such as presurgical distalizers and power are used in orthodontics which provide accurate tooth movement and customized guides for osteotomy that assist in surgical maneuvers that are as close to the 3D planning as possible [47]. Unique maxillofacial and inherently unexpected traumatic injuries can be resolved by utilizing a combination of 3D technologies that are robust, beneficial, time-saving, and reduce the menial work of material molding [48-50].

The combined effect of digitization and 3D practices in the presurgical process has allowed digitization and 3D modeling of dental arches and skeletal anatomy before planning. From low-resolution and high-rate images obtained via CT and CBCT, a high-resolution scan of occlusal arches is integral to this process [51-52]. Moreover, a composite picture of the dental-skeletal system is made possible by a CT scan of skeletal anatomy, scanned plaster models, and a reference splint with fiducial markers, via a double CBCT method, or a triple CBCT procedure has been reported [53-56]. In addition, it has been suggested by several studies that the iterative closest point algorithm should be used to position the high-resolution scans of the impression-based dental arches with appropriate craniofacial contour CT scans which eliminates fiducial marking and simplifies the process [57,58]. This study examined the accuracy of intraoral scan models (IRS) and cast scan models (CAST) on CBCT images utilizing 3D planning software. It determined the accuracy of registration based on scanning techniques and 3D programming software and concluded that registration through the PR function of 3D programming packages was significantly more precise than registration through the MR function [59]. Intraoral scanners have greatly expanded the scope of dental recordings, allowing for high-quality orthodontic occlusal data to be recorded for composite models to be loaded onto an appropriate surgical planning platform [60,61].

Patient-Specific 3D Anatomical Models

The purpose of introducing patient-specific 3D models is to provide accurate and patient-specific anatomical details for preoperative planning. These patient-specific tools reduce the operation time and preoperative planning as well as patient safety. These patient-specific, 3D-printed, anatomic models can be employed in both in and out of operation theaters for surgical planning [38]. Haptic models can be created that assist in the planning of surgical approaches by allowing cross-sectional imaging or customization of prosthetics specific to the patient’s anatomy. It reduces implantation steps and anesthesia duration [62]. Orthopedic, maxillofacial, and cardiothoracic surgeries are considered to be pioneers in applying 3D printing practices for customized prosthetics [38].

In a recent study, a comparison was made between the utility of preoperative planning with the use of a 3D-printed model and a 3D-rendered image [63]. The participants, who were surgical residents, were asked to create and review either a 3D computer model or a 3D-printed model and then formulate a preoperative plan. They scored higher on the surgical plan compared to non-3D-printed models. The researchers concluded that 3D printing may enhance the preoperative planning process for less experienced surgeons and may help develop surgical skills beyond the operating room [63].

3D printing is being used by doctors in orthodontics, maxilloplasty, and surgery to create flap designs before surgery to fix orbital hypertelorism and for maxillary reconstruction [64,65]. Additionally, the use of 3D-printed models in craniofacial surgical procedures has been utilized to treat Parry-Romberg syndrome and to plan for split calvarial bone grafting [66,67].

Virtual Surgical Planning

VSP is a minimally invasive surgical planning approach that utilizes digital clinical data to diagnose, select procedures, and plan treatment, including forecasting potential outcomes. Although the primary objective of VSP is to simplify clinical workflow, it can also be used for presurgical planning, reducing surgical time, and visualizing postoperative conditions [68].

Preoperative planning of orthognathic surgery includes the use of 2D radiographs as well as 2D model surgical procedures. However, studies [69,70] have shown that this approach has limitations, particularly for patients with significant facial deformities and asymmetries. 2D cephalometric images do not provide full information on 3D configurations. Computer-aided surgical simulations utilizing CBCT images have revolutionized orthodontic practice and have been adapted to orthognathic surgical procedures to enable cephalometric examination, surgical simulation, and splint formation [71,72].

According to a study, computer-aided techniques allowed the precise correction of malformations of the maxilla with a yaw variation, the alignment of the proximal segment and the distal segment, and the restoration of the mandibular symmetry [73]. Other studies concluded that the results of virtual orthognathic planning are aesthetically pleasing, patient satisfaction is high, the translation of the treatment plan is accurate, and the operation itself is simpler and safer [52,74]. The analyzed studies were conducted using both CT and CBCT. The obvious benefits of CT versus CBCT were improved soft tissue identification and reduced image distortion in the presence of metallic elements. Image quality, the patient’s supine position, and higher radiation doses were the key drawbacks [75,76].

Recent research has indicated that the cost and time associated with the planning and production of orthopedic occlusive splints through 3D virtual planning and the use of computed technologies is significantly lower than that associated with traditional treatment planning and the manual fabrication of splints [77-79]. In another study, Tarsitano et al. investigated the cost associated with patient-specific mandibular reconstruction plates. The study involved a cohort of patients receiving treatment for mandibular neoplasms. The population was split into two cohorts of 20 patients each, with each receiving either a traditional mandibular reconstruction or a CAD-CAM mandibular reconstruction. They concluded that computational technology for mandibular reconstructive surgery will become the standard of care for reconstructive surgery, and its cost will be covered by gains in terms of surgical time improvements, quality, and lower complication rates [80].

Discussion

Corrective jaw surgery, also referred to as orthognathic surgery or orthodontic surgery, is a surgical procedure that has evolved significantly over time. It involves the relocation of the jaw to address anomalies associated with the bite, jaw alignment, facial appearance, and respiratory function [3]. Using CAD/CAM in orthognathic surgery planning has allowed surgeons to use advanced imaging technologies such as CBCT to create 3D models of the patient’s facial bone structure, allowing for more accurate diagnosis, treatment design, and surgical prediction [10]. Intraoperative navigational systems have become increasingly popular in orthognathic surgery. These systems utilize 3D imaging and tracking technology to direct surgeons throughout the surgical process, resulting in a more accurate surgical plan, thus reducing the likelihood of errors, and improving overall surgical results [12].

The development of orthosurgical techniques, combined with the utilization of 3D printing for treatment planning, has led to an increase in the accuracy, effectiveness, and satisfaction of these procedures. As technology continues to advance, orthosurgery is expected to become increasingly sophisticated and tailored to the individual patient. In orthognathic surgery, 3D printing can be used in a variety of ways, such as replacing stone models or for the fabrication of surgical splints. Studies have identified a wide variety of advantages of 3D technologies in the treatment planning of orthognathic surgery such as drastically reducing the time required for digital planning and printing, reducing the need for multidisciplinary teams, improving the predictability of surgical outcomes, and increasing the accuracy of preoperative workups and splints [10,43,81,82].

King et al. conducted a study that revealed that the implementation of 3D technologies for oral and maxilla surgery can lead to an average reduction of 83 minutes and an expenditure of $60 per operation with the use of prefabricated surgical guides [83]. VSP has the potential to improve the surgeon’s understanding of the individual anatomy of the patient, as well as to provide a computer-driven workflow for jaw reshaping, thus replacing the traditional 2D methodologies used in orthodontic surgery. Furthermore, studies have demonstrated that 3D-planned treatment regimens can improve precision and improve results [84,85].

To precisely replicate virtual surgery during a real surgical procedure, it is essential to have an optimal intermaxillary relationship, occlusion, and face bow transfer. These transfers document the alignment of the maxilla with the hinge axis of the mandible rotation. For example, Ellis et al. [86] found an inaccuracy of almost 7 degrees when performing a face bow transfer. In a study conducted by Baily et al. [87], the average difference between the occlusal and Frankfort angle difference of the Hanau articulator was found to be 5 degrees, resulting in a 70% face bow transfer error. However, the preoperative simulation of 3D-printed plates and guides can reduce model surgery errors due to the lack of an articulator. Surgical guides and 3D-printed models are becoming more and more common in the world of facial surgery, especially in the fields of mandibular reconstruction and orthodontic surgery [88]. Patients also benefit from this technology as anatomical models enhance their knowledge of pathophysiology as well as the expected procedure, leading to better communication between patients and physicians and improved patient satisfaction [89-91]. Some authors, however, do not recommend using these 3D models on a regular basis due to the higher cost and recommend using them only for complex cases [92].

Table 2 provides a summary of all the included articles.

**Table 2:** Summary of findings.

Authors/Date	Database	Research aim(s)	Intervention/Technique	Surgical outcomes/Summary	Recommendations
Seo et al. (2021) [3]	PubMed	To explore the current trends in orthognathic surgery	3D printing	Improved surgical outcomes with a shorter duration of surgery	Improved the accuracy of osteotomy, enabled the fabrication of intermediate and final splints, and significantly shortened preoperative surgical planning with intraoperative osteotomies and fixation
Iyer et al. (2021) [4]	PubMed	To focus on the factors leading to frequently encountered conditions of acquired facial asymmetry and highlight their clinical evaluation and conservative and surgical interventions by a multidisciplinary team of clinicians	Sereophotogrammetric	Lack of exposure to radiation and lack of requirements for patient compliance	Have been promoted as effective tools for facial asymmetry diagnosis
Khechoyan et al (2013) [5]	PubMed	To describe the general surgical principles that underlie orthognathic surgery, highlighting the sequence of treatment, preoperative analysis of dentofacial deformity, surgical execution of the treatment plan, and possible complications	Virtual computer planning	Virtual computer planning promotes a more accurate analysis of dentofacial deformity and preoperative planning	It is also an invaluable aid in providing comprehensive patient education
Reyneke et al (2021) [8]	Springer	Diagnosis and planning of orthognathic surgery using different aids	3D virtual treatment planning	It behooves surgeons to continue to develop proficiency in traditional cephalometry-based treatment planning	Virtual 3D planning is another tool to aid in diagnosis and surgical planning
Levine et al (2012) [9]	PubMed	To illustrate the ease with which virtual surgery and computer-aided design and manufacturing can be used by the craniomaxillofacial surgeon to create tremendously accurate postoperative results and provide confidence with even the most complex three-dimensional bony reconstructions	The evolution of their current technique initially involved the use of stereolithographic models as templates. Pre-planning each phase of the operation including the osteotomies on the mandible and lower extremity by using staged cutting guides	Virtual surgical planning (VSP) and model design have given us the ability to visualize the surgery before it occurs, design the desired outcome, provide guides for performing the surgery, and furnish tools for confirming the match between the planned and desired outcome	Virtual planning for correction of all forms of acquired and congenital craniofacial deformities and facial syndromes can be of great benefit and produce more desirable results than traditional methods
Hammoudeh et al. (2015) [10]	PubMed	To determine whether the application and feasibility of virtual model surgery is at a point where it will eliminate the need for traditional model surgery in both the private and academic settings	VSP	The true application and potential superiority of VSP lies in the double-jaw procedures, where LeFort I and BSSO are necessary	Virtual model surgery will displace and replace traditional model surgery
Apostolakis et al. (2022) [11]	PubMed	To describe the use of the available digital technology in the workflow of CAOS and to provide insights into the advantages and limitations arising from the use of both hardware and software	Computer-aided orthognathic surgery	There is evidence that supports the use of CAOS, which is based on the lack of time-consuming preparatory steps, more accurate treatment planning, and, overall, better surgical results	There is also evidence of an increased need for training and higher costs
Anand et al. (2021) [12]	DovePress	To provide an overview of the indication of navigation in craniofacial surgeries with a focus on applied aspect, planning, and solution to the future problem	Navigation	Suggested remarkable improvements in surgical outcomes under the guidance of 3D planning and navigation	Financial expenses and a gradual learning curve are always constraining factors in surgical navigation
Farrell et al. (2020) [13]	PubMed	To evaluate the digital planning and patient-specific implants for dentofacial deformities	Digital planning and patient-specific implants	Digital planning provides perspectives of the occlusal and anatomic correction with preoperative insights that can improve intraoperative efficiency and clinical outcomes	Patient-specific implants applied for the correction of dentofacial deformities continue to evolve through the merger of advances in rigid internal fixation and digital planning
Elnagar et al. (2020) [14]	PubMed	To provide an overview of the digital workflow process for combined orthodontics and orthognathic surgery treatment starting from data acquisition (3D scanning, cone-beam computed tomography), data preparation, processing, and creation of a 3D virtual augmented model of the head	3D scanning and cone-beam computed tomography	Templates fabricated by using 3D printing fit well on the bone when surgery is performed	Although 3D virtual treatment planning of orthodontics and orthognathic surgery offers an unprecedented tool, the limitation of rendering and manipulation of the 3D data on a 2D screen may still lead to some errors in planning
Zoabi et al. (2022) [16]	PubMed	To offer perspectives on the implementation of 3D-based technologies in the field of oral and maxillofacial surgery, while indicating major clinical applications	3D printing	3D technologies have had a tremendous impact on clinical outcomes and on the way clinicians approach treatment planning. 3D printing stands out in its ability to rapidly fabricate complex structures and precise geometries	The establishment of 3D PoC facilities can bring these technologies closer to the surgeon, thereby making them easier to incorporate into daily practice and improving clinical outcomes
Hoang et al. (2016) [17]	PubMed	To learn how 3D printing has been used in surgery and how to potentially apply this technology	3D printing	There is a large array of potential applications for 3D printing. Decreasing cost and increasing ease of use are making this technology more available	The road to implementing this technology in clinical practice can initially appear daunting, with the necessary use of unfamiliar software and the large number of 3D-printing modalities available. With the use of a multidisciplinary team and rapid advancements in the field, incorporating 3D printing into a suitable application can be a highly rewarding process
Pillai et al. (2021) [18]	PubMed	To provide a brief outlook on the most recent manufacturing methods of 3D-printed objects and their current and future implications	3D printing	CT and 3D printing are paving the way to produce surgical guides; however, some of the materials used may not be autoclavable and sterilizable, thus limiting their use. In addition, accuracy is often dictated by the quality of the original scan taken by intraoral scanners, which remain inaccurate when taking full arch scans or surfaces with irregularities	New standards using the equipment will have to be defined to ensure that the patient’s standard of care, health, and safety are not compromised
Lee et al. (2021) [19]	PubMed	To develop a complete digital workflow for planning, simulation, and evaluation for orthognathic surgery based on 3D digital natural head position reproduction, a cloud-based collaboration platform, and 3D landmark-based evaluation	3D landmark-based evaluation	Orthognathic surgery outcomes performed using the digital workflow showed high accuracy for the patients	The collaboration between the surgeon and other specialists will play a central role in better planning and management of the digital workflow in orthognathic surgery
Sun et al (2013) [20]	PubMed	To present and discuss a workflow regarding computer-aided surgical planning for bimaxillary surgery and intermediate splint fabrication	Computer-aided surgical planning for bimaxillary surgery and intermediate splint fabrication	Under clinical circumstances, the accuracy of the designed intermediate splint satisfied the requirements for bimaxillary surgery	Additional studies should continue to examine the reliability and accuracy of this method in a larger series
Sahim et al. (2023) [21]	PubMed	To assess whether VSP possesses a comparative advantage over traditional surgical planning (TSP) in the context of bimaxillary osteotomy.	TSP and VSP have been used in bimaxillary osteotomy planning	VSP demonstrates superior performance over TSP in reducing the planning time for bimaxillary osteotomy, although the difference in timing during surgery is not statistically significant	The efficiency of bimaxillary osteotomy planning can be enhanced by increasing the proficiency of healthcare practitioners in utilizing recently developed technologies
Chen et al. (2021) [22]	PubMed	The objective is to assess the benefits of VSP and TSP to ascertain the potential superiority of the current VSP technique over the TSP technique for orthognathic surgery	VSP and TSP	The VSP technique demonstrated a clinically significant improvement in soft tissue prediction in the sagittal plane	The VSP technique has emerged as a viable alternative to the TSP technique in the context of orthognathic surgery, particularly with regard to frontal esthetic considerations
Barone et al. (2020) [23]	PubMed	To assess the precision of jaw repositioning in bimaxillary orthognathic surgery, a comparison was made between traditional and digital surgical planning in patients with skeletal class III	Digital surgical planning	The accuracy of jaw repositioning was significantly improved when using digital surgical planning compared to the traditional protocol	Employing digital planning is advised to attain accuracy and precision
Lonic et al. (2016) [24]	PubMed	The objective is to examine the parameters that undergo the most frequent changes during the transition from a traditional 2D plan to a 3D simulation, as well as to determine which planning parameters can be better adjusted using this method	3D simulation	3D simulation provides crucial data for precise planning in complex cleft lip/palate cases that involve facial asymmetry, which is often overlooked in traditional 2D planning	The preference lies with 3D simulation as opposed to 2D planning
Birbara et al. (2019) [25]	PubMed	The purpose of this study was to evaluate and authenticate the use of 3D modeling and printing technology for the production of patient-specific 3D models	Patient-specific 3D-printed models	The prototype method exhibited submillimeter precision for all four utilized 3D printing methods, and statistical analysis revealed a significant difference (p < 0.05) in precision among these methods	The continuous advancements in 3D modeling and printing technology could prove to be a valuable tool
Mathew et al. (2020) [35]	PubMed	The clinical implementation and advantages derived from the use of 3D models in surgical planning and execution	3D reconstruction of the deformity and pre-operative adaptation of plate	Surgical planning and execution are enhanced by 3D models in treating mid-face deficiency and extensive jaw pathologies, resulting in improved outcomes and patient satisfaction	3D-printed models are helpful in preoperative treatment planning, which significantly increases accuracy and saves time
Narita et al. (2020) [36]	PubMed	To assess and compare the operating time and amount of bleeding in two groups of patients: one group of 25 individuals who underwent surgery with the aid of a 3D model in preoperative simulations, and another group of 20 patients who did not utilize a 3D model	Desktop 3D printing technique	The development of 3D printing technology has made it feasible to obtain patient-specific 3D models at a fraction of their previous cost	In-house 3D printing techniques can be used to decrease the operating time
Mehra et al. (2011) [37]	PubMed	The aim of this study is to assess the viability of incorporating 3D stereolithographic technology into complex maxillofacial reconstructive surgery	Stereolithographic technique using 3D printing	The implementation of 3D models in oral and maxillofacial surgery has led to a substantial enhancement in the predictability of clinical outcomes when compared to treatments that do not incorporate these models	It allowed for the assessment of extensive traumatic and pathologic defects in three dimensions before surgical reconstruction
Segaran et al. (2021) [38]	PubMed	To provide the reader with insights into 3D printing and how it is used in preoperative planning	Stereolithography	Even less experienced surgeons can modify the implant shape before surgery, making it simpler	The best situation is when there is a shared comprehension of the medical and technical aspects of 3D printing until easier-to-use software becomes available
Denadai et al. (2020) [39]	PubMed	Assessing the effectiveness of computer-aided planning in cleft patients	Computer-aided planning	Achieving better surgical outcomes by reducing the burden of care	Facial aesthetics and surgical feasibility have gained valuable insights from 3D imaging and surgical simulation. The conventional method is being replaced by the surgery-first approach and two-jaw orthognathic surgery
Sun et al. (2023) [40]	PubMed	The utilization of 3D printing technology in medical education and clinical practice is highlighted by the production of low-cost and affordable 3D-printed models	3D-printed models	The educational value of 3D-printed models in medicine cannot be overstated, as they enhance the understanding of anatomy, pathology, and disease for students, graduates, patients, and their families	Clinical value is seen in personalized 3D-printed models for preoperative planning and simulating complex surgeries, leading to improved outcomes and reduced risks
Hosny et al. (2018) [41]	PubMed	The study aims to assess the accuracy of alternative techniques as existing methods overlook the finer details	Bitmap-based multi-material 3D printing	By using bitmap-based 3D printing, complex and biologically accurate functional gradients can be physically visualized, allowing for the application of this technology in new areas of medical research	Researchers are actively investigating full-color, bitmap-based printing approaches to achieve more lifelike 3D-printed representations of patient-specific anatomy
Lin et al. (2018) [43]	ScienceDirect	The benefits of incorporating 3D printing techniques in orthognathic surgery include optimal functional and aesthetic outcomes, patient satisfaction, and precise treatment plan execution	3D-printed splints, models, and implants	It provides information that can be helpful for researchers and clinicians considering the use of 3D printing techniques in orthognathic surgery	The technology can be used to make patient-specific implants and splints. It increases accuracy and adaptability
Ye et al. (2019) [44]	PubMed	The objective of this research was to determine the accuracy of 3D-printed splints produced from different dental model offsets	The Boolean operation was used to create digital splints	3D-printed splints from offset dental models provide a better fit on teeth than those from no-offset dental models. The optimal parameter for generating the splint is a 0.1 mm offset	Dental splints or guides are advanced tools used to treat dental and surgical conditions such as bruxism and temporomandibular disorders
Shaheen et al. (2018) [45]	PubMed	To enhance the existing 3D planning protocols for bimaxillary orthognathic surgery	Virtual 3D planning printing	Suggested a solution for resolving the problem of overlapping dentitions during virtual 3D planning and fabrication of digital intermediate splints for bimaxillary orthognathic surgery	Digitally 3D-printed splints were clinically accepted at an intermediate stage
Hanafy et al. (2020) [46]	PubMed	Comparing computer-aided orthognathic surgery to classic occlusal wafers for accuracy assessment	CAD/CAM splints and patient-specific osteosynthesis	This new technology made it easier to handle cases of skeletal asymmetry, reduced surgery duration and enabled trainee surgeons to perform the procedure accurately and quickly	Recommended for aspirants, but the cost is high
Thurzo et al. (2022) [47]	PubMed	This study introduces the concept and methodology of biocompatible 3D printing, along with intraoral and extraoral 3D surface scans, for custom appliances in patients with craniofacial disorders	Patient-specific 3D implants	The technique benefits infants by increasing patient compliance and accuracy, which is a main concern due to their rapid growth	Where patient compliance is not good
Costan et al. (2021) [48]	PubMed	To present the experience with using 3D printing in preoperative planning for acute mid-face trauma, an understudied area	3D-printed stereolithic models	The acute mid-face trauma setting saw favorable outcomes with the applicable 3D printing protocol	Understanding the steps for achieving the stereolithic model is crucial for adapting 3D printing to manage acute mid-face trauma
Fan et al. (2017) [49]	PubMed	To describe and assess the use of 3D printing for personalized reconstructive surgery in orbital fracture repair	3D technique-aided surgical reconstruction	In the 3D group, the average surgical duration was considerably less than in the control group. Moreover, the 3D group showed better postoperative clinical evaluation compared to the control group	The 3D printing technique is highly valuable for predicting precise fracture zones in personalized surgery, aiding accurate anatomical reconstruction for blowout orbital fracture repairs
Pang et al. (2018) [50]	PubMed	Examining the practicality of utilizing locally available 3D printing services for perioperative planning in orbital floor reconstruction with porous polyethylene	3D printing and modeling	The incorporation of a 3D-printed model decreased operative time and the duration of anesthesia. Trimming and molding defect-specific Medpor from the model easily reduces material fatigue. In addition, the model aided in educating patients and explaining the surgical procedure	The enhancement of patient care is achieved through the effective reduction of operative time and anesthesia duration
Lin et al. (2015) [52]	PubMed	A literature review on the utilization of computer-aided techniques in orthognathic surgery, encompassing surgical planning, simulation, intraoperative translation of the virtual surgery, and postoperative evaluation	3D imaging	The utilization of computer-aided methodology in orthognathic surgery offers advantages such as optimal functional and aesthetic outcomes, patient contentment, accurate execution of the treatment plan, and facilitation of intraoperative adjustments	The utilization of intraoperative guidance aids surgeons in effectively mobilizing skeletal segments to their intended position during surgical procedures, thus warranting its promotion
Tanikawa et al. (2022) [53]	PubMed	To assess the precision of dentition superimposition on CBCT images by employing palatal mucosa, both with and without the application of barium sulfate coating	Intraoral digital models	The errors observed in CBCT images acquired with barium sulfate were markedly reduced compared to uncoated images	A novel and non-invasive technique was developed to precisely overlay an intraoral digital model onto CBCT images using barium sulfate-coated palatal mucosa
Barone et al. (2013) [54]	PubMed	To introduce a multi-modal framework that enables the fusion of diverse digital techniques, resulting in the creation of a comprehensive 3D virtual maxillofacial model. This model seamlessly integrates a photorealistic face, facial skeleton, and dentition	The aim of this study is to establish a superimposition method on the lower arch utilizing 3D CBCT images and orthodontic 3D digital modeling	The technique ensures precise placements among distinct anatomical tissues via pairwise fusion processes, as the procedure is guided and controlled by ground truth references	Full automation of these activities is necessary for the implementation of 3D virtual imaging in daily practice
Park et al. (2012) [55]	PubMed	To develop the superimposition method on the lower arch by utilizing 3D CBCT images and orthodontic 3D digital modeling	3D imaging	The surface superimposition method produced relatively more consistent coordinate values	Surface superimposition proved to be the simpler and more reliable method for evaluating 3D changes in the lower arch
Lin et al. (2015) [56]	PubMed	To evaluate the reliability of point-based superimposition of a digital dental model onto a 3D CT skull with undamaged dentition	3D CT	Achieving clinically acceptable accuracy is possible through the utilization of a direct point-based method for superimposing a digital dental model onto a 3D CT skull	Not applicable
Kim et al. (2010) [57]	PubMed	To assess the precision of the fusion of CT-derived bone models and laser-scanned dental models using sequential point- and surface-based markerless registration for the formation of a digital maxillofacial dental model	3D models	Accurate integration of the maxillofacial dental composite model can be achieved without the use of fiducial markers, despite the differing resolutions of the CT and dental models	Not applicable
Noh et al. (2011) [58]	PubMed	To assess the registration errors associated with the integration of laser-scanned dental images into CBCT scan data, as well as to investigate the impact of the registration area on the registration accuracy	3D imaging.	The findings of this study suggest that the accuracy of integrating laser-scanned dental images into maxillofacial CBCT images is enhanced when a larger registration area is utilized	Minor details like tooth structure may get missed, which may be necessary for the treatment
Park et al. (2020) [59]	PubMed	To utilize 3D planning software to register intraoral scan (IS) models and cast scan (CS) models onto CBCT images. Furthermore, to assess the accuracy of registration based on scanning methods and 3D planning software	Intraoral scan models and cast scan	The accuracy of registration using the PR function of the 3D planning software packages was notably superior to that of registration using the MR function	Not applicable
Nilsson et al. (2016) [60]	PubMed	To create a 3D model of the craniomaxillofacial region and utilize intraoral digital scanning for the precise positioning of the lower jaw in centric relation, thereby obviating the necessity for plaster casts and model surgery	3D model	It reduces the lab work in recording virtual bites. No casts and models are required	Recommended for digital sharing of data without transferring physical impressions
Waard et al. (2016) [61]	PubMed	This study evaluates the feasibility of adding a detailed dentition surface model to the 3D virtual skull using intraoral scanning compared to the triple scan procedure	3D imaging	Intraoral scans offer a precise depiction of the dental arches in comparison to AlgiNot-dental casts and can be combined with CBCT scans	The proposed method provides benefits and demonstrates clinical feasibility in the integration of intraoral scans into CBCT scans, thereby enhancing orthognathic surgery planning
Zheng et al. (2016) [63]	PubMed	To compare 3D-rendered images and 3D-printed models for treatment planning	3D imaging and model	3D-printed models improve the quality of surgical trainee’s preoperative plans	3D-printed models are better than 3D-reconstructed images
Engel et al. (2015) [64]	PubMed	Surgical correction was planned using 3D printing modeling in severe orbital hypertelorism of an 11-year-old boy	3D model	This approach enabled a reduction in surgical time, precise planning of osteotomy locations, and pre-contouring of osteosynthesis materials	3D models are very helpful tools in planning complex craniofacial operative procedures
Nkenke et al. (2014) [65]	PubMed	The primary goal of the procedure is to restore the patient’s normal function and appearance	3D model	The prefabrication of the vascularized free fibula graft enabled the simultaneous occurrence of prosthetic rehabilitation and bony maxillary reconstruction	Not applicable
Chai et al. (2015) [66]	PubMed	To explore the utilization of a 3D scanning and printing system in combination with an anterolateral thigh dermal adipofascial flap for the management of Parry-Romberg syndrome and facial soft tissue reconstruction	3D-printed models	Models were generated through the use of 3D printing to map areas of soft tissue deficiency. The design of anterolateral thigh dermal adipofascial flaps was developed using 3D models of soft tissue insufficiency. All flaps survived	For adaptation of flaps and grafts
Mendez et al. (2015) [67]	PubMed	To assess the viability, expense, and production timeline of personalized skull models produced via an in-office 3D printer for craniofacial reconstruction	VSP using 3D printing	The feasibility of VSP “in office” 3D printing has been demonstrated, offering a more cost-effective and time-efficient approach to creating surgical models and guides	Intraoperative efficacy can be improved with low-cost technology
Singh et al. (2021) [68]	PubMed	To summarize the current state of VSP	3D model	These approaches offer insights that can contribute to the advancement of tailored surgical procedures and intelligent medical devices with utmost accuracy and precision	For precision and accuracy
Ho et al. (2017) [69]	PubMed	To assess surgical plan modification after 3D simulation	3D printing	The implementation of 3D computer-aided surgical simulation contributes to the enhancement of planning for patients with complaints of facial prognathism and asymmetry	Better outcomes than the conventional methods
Seres et al. (2014) [70]	PubMed	This report showcases a case of facial asymmetry caused by computerized simulation surgery instead of manual model surgery, resulting in a virtual wafer splint fabrication	3D-printed splint	The facial symmetry experienced a notable improvement, leading to the attainment of a stable occlusion. The benefits of computer-aided surgical planning and 3D rapid prototyping are demonstrated by this intricate case, which involves the correction of facial asymmetries	Recommended for complex cases
Lee et al. (2016) [71]	PubMed	To evaluate preoperative planning using a 3D-printed model	3D model	Guidance for bimaxillary orthognathic surgery included intraoperative visualization and quantification of deviations. Simulated skeletal models and landmarks enhance conventional navigational surgery for bone repositioning in the craniomaxillofacial area	Not applicable
Adolphs et al. (2014) [72]	PubMed	The retrospective assessment involved comparing predictions and surgical results to evaluate the potential and feasibility of virtual craniomaxillofacial surgery as an additional planning tool	3D-printed models	Virtual craniomaxillofacial planning has proven to be a beneficial adjunctive planning tool for determining the ideal surgical approach in individualized treatment concepts	The integration of traditional 3D models with virtual simulation has enhanced the efficiency of planning and implementation of craniomaxillofacial corrections
Hsu et al. (2013) [73]	PubMed	The objective of this prospective multicenter study was to evaluate the precision of a computer-aided surgical simulation (CASS) protocol for orthognathic surgery	Computer-generated surgical splints	The CASS protocol enables the accurate and consistent transfer of the computerized plan to the patient, facilitating precise alignment of the maxilla and mandible during surgery	The accuracy of repositioning the chin segment is significantly enhanced by utilizing the computer-generated chin template, as opposed to relying solely on intraoperative measurements
Lin et al. (2015) [74]	PubMed	To develop a technique for creating personalized positioning guides to facilitate the translation of virtual plans into real orthognathic surgery, as well as to assess the practicality and accuracy of the developed technique	Computer-aided positioning guide	The proposed customized positioning guides offer practicality and reliability in the translation of virtual plans to real-life surgical interventions. Additionally, these guides have enhanced the efficiency and outcomes of surgical procedures	The design of this approach is straightforward, the fabrication is cost-effective, and it is particularly convenient to use
Resnick et al. (2016) [77]	PubMed	This study aimed to evaluate the cost difference between VSP and 3D printing of splints versus 2D cephalometric evaluation, model surgery, and manual splint fabrication	VSP and standard planning	The findings of this study suggest that utilizing VSP for bimaxillary orthognathic surgery results in reduced time and costs compared to standard planning for the analyzed cases	VSP is economical as well as more accurate
Steinhuber at al. (2018) [78]	PubMed	To assess and contrast the duration of VSP versus conventional surgical planning (CSP) in orthognathic surgery, taking into account the surgical procedure, personnel, and work setting	The treatment approach involves manual splint fabrication for CSP and CAD/CAM splint fabrication for VSP	Office-based VSP for orthognathic surgery was significantly faster for single- and double-jaw surgery	Not applicable
Park et al. (2019) [79]	PubMed	To analyze the time and cost variations between CSP and VSP in orthognathic surgery	Surgical stents were fabricated through manual and 3D printing.	The time investment in VSP in this study was significantly smaller than that in CSP	VSP
Tarsitano et al. (2016) [80]	PubMed	To evaluate the cost generated by the management of this technology	CAD/CAM technologies	CAD/CAM technology is projected to emerge as a prevalent approach to mandibular reconstruction, as its expenses are anticipated to be compensated by the benefits of reduced surgical time, enhanced reconstruction quality, and fewer complications	The application of CAD/CAM technologies in maxillofacial bone reconstruction offers the advantages of increased precision, reduced morbidity, and shorter operative time
Kim et al. (2011) [81]	PubMed	To present the clinical experience concerning the production and precision of digitally printed wafers for maxillary movement during bimaxillary orthognathic surgery	Digital model surgery	The accuracy levels of wafers produced via digital model surgery (DMS) were akin to those achieved through manual model surgery, albeit marginally lower than those solely produced by DMS	Manual and digital model studies have similar outcomes
Chaudhuri et al. (2022) [82]	PubMed	To identify the value proposition, creation, capture, and provision of value to users by healthcare 3D printing service providers, as well as the required resources and capabilities for value co-creation by the clinical team	3D printing	Providers of 3D printing services and hospital surgical teams have the opportunity to collaborate and leverage their resources to enhance capabilities and create value through their interactions	As hospitals are at the initial stages of incorporating 3D printing for surgical procedures, 3D printing service providers are capitalizing on their exploitative capabilities, while the surgical team is showcasing a combination of explorative and exploitative capabilities to actively engage in the co-creation process and generate value
King et al. (2018) [83]	PubMed	To compare the intraoperative time and operating room costs among patients with mandibular fractures who underwent conventional adaptation and fixation versus those who were treated with preadapted plates generated from on-site 3D-printed models	3D modeling and printing	The utilization of 3D printers for the fabrication of models in prebending maxillofacial reconstruction plates is correlated with a reduction in both operating room time and costs	Employing an on-site 3D printer involves minor start-up and usage expenses, leading to a noteworthy reduction in operating room time, which continues to be one of the most expensive elements of facial trauma care
Heufelder et al. (2017) [84]	PubMed	To evaluate the efficacy of a recently developed approach for waferless maxillary positioning in bimaxillary orthognathic surgery, employing customized surgical guides and patient-specific osteosynthesis implants	Customized surgical guides and patient-specific osteosynthesis fabricated using CAD/CAM technology	Achieving waferless maxillary positioning in dentofacial deformities can be accomplished with exceptional precision by employing CAD/CAM patient-specific implants and surgical guides to translate the virtual simulation into surgical practice	The implementation of this technique has the capacity to alter the current approach to maxillary positioning in clinical routine
Kim et al. (2023) [85]	PubMed	This research seeks to critically assess the progression and limitations of traditional approaches in orthognathic and oral maxillofacial surgery, while also exploring the potential advantages of integrating advanced technological tools, such as 3D technology, into surgical procedures	3D printing	Not applicable	The combination of 3D printing and VSP serves as a catalyst for transforming surgical planning and implementation. This is accomplished by offering tactile 3D models for visualization and planning, as well as precisely designed surgical guides for accurate execution. Professionals are required to attain the necessary skills for utilizing the software employed in the design and creation of 3D-printed models and surgical guides
Bailey et al. (1981) [87]	PubMed	The study compared relationships between the occlusal plane and the Frankfort plane using radiographs and articulator transfers	Radiographs	No method shows a definite advantage in transferring the Frankfort plane to the articulator	The Frankfort plane-maxillary occlusal plane relationship that exists in a subject is not transferred to the Hanau articulator with the two third points of reference studied
Amundson et al. (2020) [88]	PubMed	The emergence of reliable tools such as VSP, surgical navigation, intraoperative imaging, and customizable implants have made them important topics of discussion in implant surgery and orthognathic surgery	3D printing, 3D modeling	Not discussed	The implementation of VSP, surgical navigation, intraoperative imaging, and customizable implants has proven to be effective in implant surgery and orthognathic surgery, and their adoption is increasing in the trauma setting
Yamada et al. (2014) [89]	PubMed	The usefulness of mandibular reconstructions employing a custom-made titanium mesh (Ti-mesh) tray and particulate cancellous bone and marrow was evaluated	3D printing	In six out of nine patients, there was remarkable new bone formation observed, with radiological results meeting expectations. Four patients experienced complications. The complications encompassed fracture of the Ti-mesh, exposure of the Ti-mesh in the oral cavity, and delayed infection	To avoid fracture of the Ti-mesh tray, it is advisable to consider a combination repair involving a titanium reconstruction plate or the creation of a reinforced Ti-mesh tray for future cases with long-span defects, such as those in the chin area
Gerbino et al. (2015) [90]	PubMed	To examine surgical outcomes in patients with craniofacial defects who underwent primary and secondary reconstruction using PEEK patient-specific prostheses created with CAD/CAM	Patient-specific implants	No complications	The accurate restoration of the complex 3D anatomy of the craniofacial region is made possible through the use of PEEK CAD/CAM implants
Farrell et al. (2014) [91]	PubMed	To gauge the increased efficiency through virtual planning	CASS	Research has proven the accuracy of VSP and its ability to improve clinical outcomes compared to the traditional model	VSP allows for preoperative understanding of the surgery and the use of cutting jigs/guides and templates can reduce intraoperative surgical inaccuracies

## Conclusions

The utilization of 3D printing models for oral, maxillofacial, orthognathic, and other surgeries is becoming increasingly popular due to their safety, reduced trauma, and shortened treatment times. Furthermore, 3D printing enables a more expeditious and accurate assessment of surgical, preoperative, and postoperative procedures, allowing for more efficient and accurate treatment planning. 3D modeling for preoperative planning improves the 3D view of the planned operation. It enables pre-adaptation of surgical tools such as fixation plates, shortening the operation time and improving accuracy. The utilization of 3D-printed aids enables the precise re-creation of anatomical relationships and the prompt restoration of functions during orthognathic surgeries. Additionally, as these technologies do not need to be adjusted in the operating room, the implants are strong and can handle all kinds of physical activity. 3D printing is becoming more and more popular, and we can expect to see several new treatments made with 3D printing in the near future.
